# Variations in Accumulated-Training Load Parameters and Locomotor Demand with Consideration of Puberty in Elite Young Soccer Players

**DOI:** 10.3390/biology11111594

**Published:** 2022-10-30

**Authors:** Hadi Nobari, Hamed Kia shemshaki, Okan Kamiş, Rafael Oliveira, Pablo Prieto González, Elena Mainer-Pardos

**Affiliations:** 1 Department of Exercise Physiology, Faculty of Educational Sciences and Psychology, University of Mohaghegh Ardabili, Ardabil 56199-11367, Iran; 2 Department of Motor Performance, Faculty of Physical Education and Mountain Sports, Transilvania University of Braşov, 500068 Braşov, Romania; 3 Faculty of Sport Sciences, University of Extremadura, 10003 Cáceres, Spain; 4 National Institute of Physical Education of Catalonia—Lleida Campus, Health and Management Partida la Caparella, University of Lleida, 25194 Lleida, Spain; 5 Faculty of Sports Sciences, Aksaray University, 68100 Aksaray, Turkey; 6 Institute of Health Sciences, Gazi University, 06500 Ankara, Turkey; 7 Sports Science School of Rio Maior–Polytechnic Institute of Santarém, 2040-413 Rio Maior, Portugal; 8 Research Center in Sport Sciences, Health Sciences and Human Development, 5001-801 Vila Real, Portugal; 9 Life Quality Research Centre, 2040-413 Rio Maior, Portugal; 10 Health and Physical Education Department, Prince Sultan University, Riyadh 11586, Saudi Arabia; 11 Health Sciences Faculty, Universidad San Jorge, 50830 Zaragoza, Spain

**Keywords:** aerobic power, training strain, training monotony, football, maturation, youth

## Abstract

**Simple Summary:**

Scientific research has demonstrated that puberty status has a crucial influence on soccer players, and their energy demands are primarily determined by their aerobic capacity. In our investigation, we examined the relationships between maturation variables such as peak height velocity (PHV) and maturity offset with variations in accumulated-training load parameters and locomotor demands in elite young soccer players; the aim was to predict the percentage of changes in their performance ability with adjustments to the training load parameters considering maturation variables. Our study’s main findings revealed a link between accumulated-training load parameters and locomotor demands. In addition, the acute:chronic workload, training monotony, and PHV values explained aerobic power performance. This study offers new valuable insights into the practice of accumulation and motor demand in young elite players.

**Abstract:**

The study’s purposes were to examine the associations of training load parameters with locomotor demand and puberty status in elite young soccer players and to predict the percentage of changes in their performance ability with adjustments to the training load parameters, using multivariate regression analysis, while considering PHV and maturity offset. Seventeen male players (15–16 years old) participated in this study. Anthropometrics, body composition, maximal oxygen consumption (VO_2max_), and puberty status (for calculating PHV) and maturity offset were assessed. The results demonstrated substantial differences between the PHV, VO_2max_, and load parameters (acute and chronic workload (CWL)) over a soccer season. A substantial relationship existed between the workload parameters (VO_2max_, CWL, and training monotony (TM)) and maturity offset. All of the variables, except for training strain, demonstrated significant variances in relation to the differences between the first and second halves (*p* < 0.05). Aerobic performance can be estimated using the CWL, TM, and maturity offset values (R^2^ = 0.46). On the contrary, aerobic power performance can be explained using the acute:chronic workload, TM, and PHV values (R^2^ = 0.40). In conclusion, the biological maturity state of young soccer players has a substantial impact on their functional potential. Variations in accumulated load contribute significantly to aerobic resistance, whereas weight and height contribute significantly to sprint and vertical-jump performance, respectively.

## 1. Introduction

Soccer can be performed by both adults and children and is the most widely recognized sport in the world [1]. Various features influence performance, including technical, tactical, physiological, and mental factors [2]. During training and matches, these locomotor activities (e.g., total distance, high-speed running, accelerations, and decelerations) compound the total physical load throughout the season [3]. Soccer players’ energy demands are primarily determined by their aerobic capacity [1]. High-level skills such as jumping, kicking, and tackling are anaerobic, even though aerobic metabolism supplies a football game’s basic physiological and metabolic processes. Therefore, anaerobic demands are also crucial in soccer [4].

Moreover, high levels of anaerobic and aerobic capacity are necessary for soccer players to produce powerful, match-specific moves such as sprinting, jumping, and accelerating [5,6]. During the preseason, it is crucial to improve aerobic fitness. Although the match itself can improve the oxygen transport system, it does not do so at a fast-enough rate to produce the ideal condition for physiological improvement. As a result, in the pre-competitive season, preparation is likely to include more structured fitness and conditioning [7]. Studies on youth soccer populations’ match running performance have increased over the past ten years, offering data that could help design physical conditioning programs [8,9].

Soccer matches involve several high-intensity running phases and sprints mixed with aerobic-type recovery activities [1,10]. In addition, the anaerobic system is important during the match and is key to successfully executing kicks, explosive effects, and changes in direction [1]. It is well known that training methods can strengthen young players’ neurological and muscular condition associated with aerobic and anaerobic power by developing the capacity to tolerate higher levels of hydrogen ions [11]. Therefore, with biological development, these physical capacities increase with age [12].

Maturation is the term for changes in the body’s anatomy and functionality as it develops, such as the transition from cartilage to bone in the skeleton, menstruation, or the growth of pubic hair [13]. This process starts with a height increase and continues with growth. After this spurt, there is an eventual cessation in height until the end of growth [14]. Another non-invasive and predictive method is used to determine the onset and subsequent progression of the growth spurt in puberty (i.e., maturity offset), also known as the period before or after PHV [15]. Scientific research has shown that maturity status significantly impacts the discovery and selection of young soccer players with talent [16,17]. In single-year chronological age groups of males from late infancy to mid-puberty, fat-free mass and several other physical fitness factors, including endurance, muscular strength, power, speed, and aerobic power, are all associated with greater biological maturity (approximately 9–16 years) [13,18]. In practice, late-maturing soccer players face an unequal battle, as early-maturing players are typically more prominent, perform better than their late-maturing counterparts, and are chosen by trainers for selection [19].

On the other hand, the effect of maturation on physical capacities among soccer players has been investigated by sports scientists [20,21]. For instance, Eskandarifard et al. [22] observed significant changes in maturation levels, insulin-like growth factor-1 (IGF-1), aerobic capacity, and power performance levels depending on the play duration and the maturation groups’ level. When playing time and maturity level were combined, substantial changes in maturity offset and power performance were discovered [22].

Acute workload (AWL, the level of load in one week) or chronic workload (CWL, the level of load in four weeks) are simple and low-cost parameters for trainers and practitioners to calculate the distribution of sessions per week and workload implications [23]. Moreover, TL monitoring examines individual answers to training in team sports. Recent research has observed that TL is usually higher during the preseason than in the competitive season in soccer [24]. By contrast, especially in youth players, physical performance improves during the competitive season, while TL declines because of their growth and maturation. Training load and physiological changes can also impact a player’s performance over a season [25]. Previous research has examined the correlation between soccer training load and injuries [26], as well as player fitness [17,25], recovery, and tiredness before and after matches [24]. These data provide meaningful information to practitioners regarding the importance of training load and the effects of workload during an entire season. 

The literature on top youth football players has examined the relationship between changes in training load and maturational parameters, and numerous articles have been published [8,17,25,27,28]. However, differences in accumulated-training load parameters and locomotor demand regarding maturations have not been well studied in young elite players throughout the competition season [28]. Therefore, coaches and practitioners must have access to these data to comprehend weekly session distribution and workload levels properly. 

Given the importance of TL and locomotor demand with consideration of maturity among elite young soccer players, the primary purposes of this research were as follows: (i) We aimed to describe the relationships between maturation variables (e.g., PHV and maturity offset) with variations in accumulated-training load parameters and locomotor demands in elite young soccer players. (ii) The main novelty of this study was the prediction of the percentage of changes in performance ability with adjustments to the training load parameters using multivariate regression analysis while considering PHV and maturity offset. Additionally, a second complementary aim was to compare locomotor changes and TL parameters between the first and the second half of the season. In light of the main aims, it was predicted that locomotor changes in elite young soccer players over the course of a season might be explained by accumulated-training load and maturation. 

## 2. Materials and Methods

### 2.1. Participants

Seventeen elite youth male soccer players between 15 and 16 years of age voluntarily enrolled to participate in this investigation after providing informed consent (age: 15.5 ± 0.4 yr.). They participated in the regional league of Iran. The University of Mohaghegh Ardabili ethics committee authorized the study (11.04.2020), which adhered to certain standards. The legal guardians of the athletes and participants gave their written consent in advance of their voluntarily participation in this study after a detailed description of the study procedures, the experimental protocol, and the potential risks and benefits. The players were in the following positions: six defenders, three wingers, six central midfielders, and two forwards. Based on previous studies that highlighted large-to-extensive correlations between workload parameters with maturation and performance variables in young soccer players [22,29], a priori correlation (point-biserial model) analysis to calculate sample size power was conducted with the following information: α err prob = 0.05, power (1-β err prob) = 0.80, and effect size = 0.55. The result showed that sixteen participants would be required to achieve 80.5% of the actual power. For the calculation of sample power, G*Power software (University of Düsseldorf, Düsseldorf, Germany) was used.

### 2.2. Study Design

A descriptive longitudinal study was used to observe the entire match-monitoring season by playing position in a U16 soccer team during the 2021/2022 season. Matches were monitored daily for fifteen weeks and divided as follows: first half of the season: week (W) 1 to W8, and the second half of the season: W9 to W15. Descriptive data on matches and training sessions during the season are shown in Table 1. 

### 2.3. Procedures

Two consecutive days were used to evaluate the players. On the first day, anthropometrics, body composition, maturation status for calculating PHV, and maturity offset were assessed during the morning. The 15–30 intermittent fitness test (15–30 IFT) was performed on the second day. The first and second stages of the evaluations were completed at 8–11 am at 12–16 °C and 27–35% humidity in a natural-grass soccer field [29]. All players were previously familiarized with the aerobic power test.

### 2.4. Anthropometrics and Maturation

Height and sitting height were measured with a precision of 5 mm using a stadiometer (Seca model 213, Hamburg, Germany). Using a digital scale (Seca model 813, Birmingham, UK), weight was calculated to the nearest 0.1 kg. Age at PHV and maturity offset were predicted using the information collected above and the equation proposed by Mirwald et al. [15]. 

### 2.5. Monitoring Training Load

Each player was asked to individually provide their rated perceived exertion (RPE) in arbitrary units (A.U.): Using the question “How did you feel about the intensity of the training?”, RPE was assessed using Borg’s CR-10 scale ranging from 0 to 10 half an hour after each training session [30]. Players were previously familiarized with the scale during two years at the club. The scores provided by the players were also multiplied by the training duration to obtain the s-RPE. A total load of daily training during the week was considered the weekly acute workload (AWL); the following formulas [24,31] were calculated for weekly chronic workload (CWL) (the rolling exponential of the average accumulated-training load of training sessions experienced in the previous three weeks); acute chronic workload ratio (ACWR) (calculated by dividing the acute workload (1-week rolling workload data) by the chronic workload (the rolling 3-week average workload data)); weekly training monotony (TM) (weekly AWL ÷ standard deviation (SD) of this week’s AWL); and eventually, weekly training statin (TS) (weekly AWL × weekly TM).

### 2.6. Locomotor Demand Test

Locomotor demand was determined as aerobic fitness via the 15–30 IFT for maximal oxygen uptake (VO_2max_). The 15–30 IFT was evaluated as described elsewhere [32]. The test was terminated when subjects could not maintain the beep rate of the protocol. The formula used to calculate VO_2max_ (mL·kg^−1^·min^−1^) was 28.3 − (2.15 × 1) − (0.741 × 16-years) − (0.0357 × body mass) + (0.0586 × 16-years × final running speed) + (1.03 × final running speed.

### 2.7. Statistical Analysis

Normality was verified using the Shapiro–Wilk’s test. Data are presented as mean and SD. Pearson (AWL in the first and second half, CWL in the second half, ACWL and TL in the first half, PHV and maturity) and Spearman (TM in the first and second half, CWL in the second half and ACWL in the first half) correlations were examined for the WL parameters, maturity, and PHV. Additionally, range intervals for the magnitude of correlations were added (e.g., r = 0.10 to 0.29—small; r = 0.30 to 0.49—moderate; r = 0.50 to 1—strong) [33].

In addition, a dependent *t*-test or Wilcoxon test (non-parametric) with a 95% confidence interval (CI) was used to calculate differences within the first half vs. the second half for the WL parameters and aerobic power variable. The effect size was calculated using Cohen’s *d*. The effect size values were then classified as 0.20—small, 0.6—moderate, 1.2 —large, and 2.0—very large [34]. Moreover, multiple linear regression analysis of the percentage of change in aerobic power, with variations in maturity variables (PHV and maturity offset), were carried out. Finally, the Akaike information criterion (AIC) was calculated to support inferences about the model’s suitability. SPSS (SPSS 25.0; IBM SPSS Inc., Chicago, IL, USA) and GraphPad Prism 8.0.1 (GraphPad Software Inc, San Diego, CA, USA) were used for all statistical calculations. 

## 3. Results

Of the correlations between maturity offset and aerobic power parameters (Table 2), the most important was VO_2max_ in the first half, related to VO_2max_ in the second half (r = 0.85; *p* ≤ 0.01). Additionally, AWL during the first half was associated with TM during the first half (r = 0.47; *p =* 0.05). Additionally, there was a relationship between CWL in the first half and ACWR in the first half (r = −0.59; *p* ≤ 0.01) and TS in the first half (r = 0.40; *p*= 0.04). There were associations between CWL in the second half and ACWR during the second half (r = 0.41; *p* = 0.05) and TS during the second half (r = 0.51; *p* ≤ 0.01). Additionally, there was an association between ACWR in the first half and TS in the first half (r = −0.37; *p*= 0.03). Finally, TM during the first half was related to TS in the first half (r = 0.96; *p* ≤ 0.01).

Between VO_2max_ during the first half and VO_2max_ during the second half, there were significant positive associations (r = 0.85; *p* ≤ 0.01) (Table 3). There were correlations between AWL in the first half and TM in the first half (r = −0.54; *p* ≤ 0.01) and TS in the first half (r = 0.34; *p* = 0.03). Likewise, AWL during the second half was associated with CWL in the second half (r = 0.62; *p* ≤ 0.01), ACWR during the second half (r = 0.83; *p* ≤ 0.01), and TS in the second half (r = 0.34; *p*: 0.05). Additionally, CWL during the first half was associated with ACWR in the first half (r = 0.53; *p* ≤ 0.01), TM during the first half (r = 0.32; *p* = 0.05), and TS in the first half (r = 0.54; *p* ≤ 0.01). In addition, CWL during the second half was related to ACWR in the second half (r = −0.53; *p* ≤ 0.01) and TS in the second half (r = 0.54; *p* ≤ 0.01). Finally, ACWR was related to TS during the first half (r = 0.34; *p* = 0.04).

**Figure 1:**
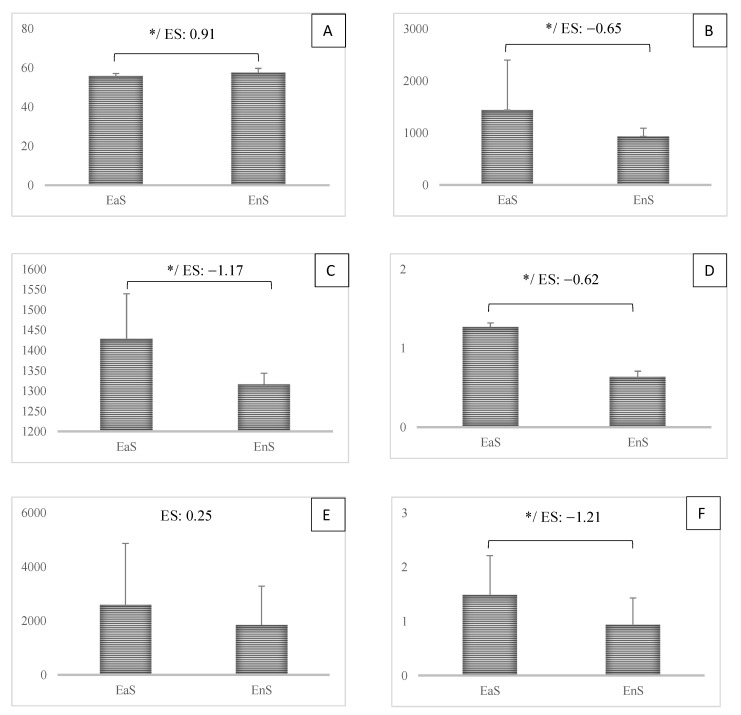
Comparison of 1st- and 2nd-half seasons in the workload parameters and aerobic power. ES: effect size. * *p* < 0.05. (**A**) VO2max: maximum rate of oxygen consumption; (**B**) AWL: the accumulated acute workload in the season; (**C**) CWL: the accumulated chronic workload in the season; (**D**) ACWR: the accumulated acute chronic workload ratio in the season; (**E**) TS: the accumulated-training strain in the season; (**F**) TM: the accumulated-training strain in the season.

Figure 1 shows a comparison between the first- and second-half workload and aerobic power results. All variables, except for TS (*p* > 0.05; ES: 0.25), showed statistically significant variations between the first and second halves (*p* 0.05; ES: −1.21 to 0.91).

**Figure 2:**
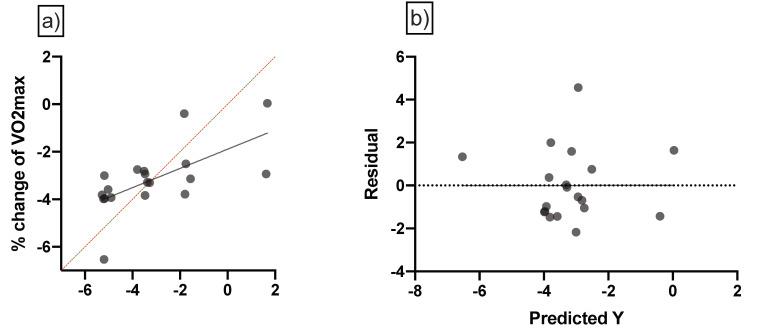
Multiple linear regression analysis was calculated to predict the percentage of change in fitness levels (**a**) VO_2max_ based on accumulated workloads. and PHV in the soccer players. Also. (**b**) residual plot was calculated to predict the percentage of change in VO_2max_ levels; the difference between the actual value of the dependent variable and the value predicted by the residual provided. PHV = Peak height velocity; VO_2max_: maximum rate of oxygen consumption.

In order to predict the percentage change in aerobic power based on maturity offset, multiple linear regression analyses were carried out (Table 4 and Figure 2). Aerobic power revealed that there were significant differences (F (3, 15) = 4.34, *p* = 0.02), with an R^2^ value of 0.46. Participants showed good predictions for aerobic power; (Y) is equal to Beta0 + Beta1 (CWL) + Beta2 (TM) + Beta3 (maturity offset). 

**Figure 3:**
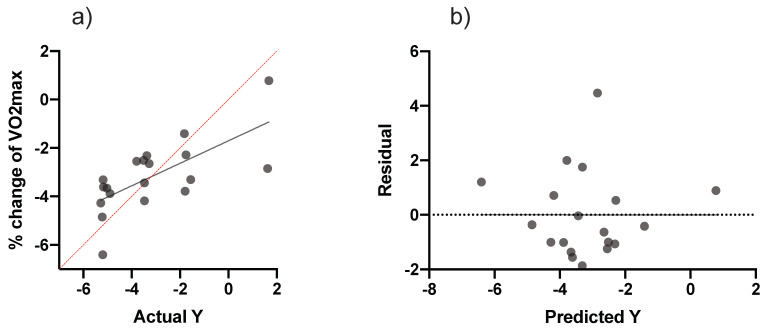
Multiple linear regression analysis was calculated to predict the percentage of change in fitness levels (**a**) VO_2max_ based on accumulated workloads and maturity offset in the soccer players. Also, (**b**) residual plot was calculated to predict the percentage of change in VO_2max_ levels; the difference between the actual value of the dependent variable and the value predicted by the residual provided. VO_2max_: maximum rate of oxygen consumption.

In order to predict the percentage change in aerobic power based on PHV, multiple linear regression analyses were carried out (Table 5 and Figure 3). Aerobic power revealed that there were significant differences (F (3, 15) = 3.40, *p* = 0.04), with an R^2^ value of 0.40. Participants showed good predictions for aerobic power; (Y) is equal to Beta0 + Beta1 (ACWR) + Beta2 (TM) + Beta3 (PHV). 

## 4. Discussion

This study’s primary objective was to assess the associations between training workload variables and variations in locomotor demand in the first and second half of the season in talented young soccer players. We hypothesized that the accumulated-training load and maturity might explain variations in the season’s locomotor demands for top-level young soccer players. The results supported our predictions. The major findings were that there is a link between accumulated-training load parameters and locomotor demand, and we found significant improvements in the second half. In addition, significant correlations were found between the locomotor demand and workload parameters. Finally, the percentage of change in aerobic power can be estimated using CWL, TM, and maturity offset and using ACWR, TM, and PHV during the season in elite young soccer players.

It is a fallacy that traditional methods are often used to identify and choose soccer talent. Some players mature quickly and are the best in both the short and long term [35,36]. Nevertheless, it is unfortunate that players with premature puberty are selected more than late-maturing players due to physical characteristics such as taller and stronger bodies, meaning that these more minor teammates are deprived of selection [13,36]. It has also been found that people with premature maturation and PHV run longer distances [35]. On the other hand, people with premature puberty and taller heights can run faster than their peers with late puberty [37]. Despite the dichotomy in the research background, it seems that players with early maturity and taller heights have a better chance of succeeding in achieving elite player status [38].

The results of this study reveal a link between accumulated-training load parameters and locomotor demand, and we found significant improvements in the second half. However, more pronounced levels were connected to HPV between the first and second half. Teenage physical measurements, functional ability, and skills change at different puberty times [39]. Indeed, velocity statistics suggest that pre-PHV increases are highest in boys [40], but strength and power tests are highest after PHV [13,41]. Another critical issue is the differences in the opportunities given to players with early and late maturity when playing and being selected for a match. Currently, the process of selecting players is such that stronger and taller players (early players) are selected to play because these players are at or near the height of their development [15]. Furthermore, they contribute more to winning matches; this contrasts with the developmental process for late-maturing players, and these decisions increase their likelihood of quitting soccer [37,42]. 

According to research reports, the development of the peak of physical fitness in children often coincides with the peak of height development, which occurs around the age of 14 years [42]. A study of 11- to 15-year-olds discovered that peak height occurred between 13 and 14 and coincided with peak aerobic capacity, speed, and agility [42]. However, in similar studies, researchers showed that the peak of power growth occurs between the ages of 15 and 16 [43,44]. So, based on the mentioned data, we chose this age range as the selection criterion based on the above statistics. Based on the previous findings, significant connections between aerobic power, speed characteristics, and maturity state were found among the target soccer group’s periods. Regression models using accumulated load, baseline levels, and PHV as predictors were also used to attempt to explain variations in fitness levels throughout the season. Significant predictors of aerobic power levels (VO_2max_ and HRrest) were discovered during this test, but not for the speed variables (i.e., linear sprint, medium and short), while for HR, HRmax was only reported as significant during the racing season [13].

According to our results, players with higher levels of maximal oxygen consumption appear to be better at power-related performance. Therefore, maximum oxygen consumption is a determining factor in the selection of players for the match and in players spending more minutes in the match [39,45]. Furthermore, people with premature puberty are predicted to have more neurological and structural stimuli, which maximizes existing physical characteristics. Consequently, players with early maturity will perform better than players with late maturity. Players with early maturity have better aerobic capacity, speed, strength, and power than players with late maturity [46,47]. However, late-maturing soccer players seem to have better balance and body control than their early-maturing counterparts. Some differences in bio-motor abilities between players with early and late puberty have been mentioned [48].

Despite the current study’s novelty, various restrictions must be considered: (1) The number of young elite soccer players participating was minimal; by adding more teams, it is possible to sample more representatives of the young talent population. However, the number of participating players could be another limitation. (2) We failed to use daily player heart rate (HR) data and the Global Positioning System for each player, which could have identified more specific elements. (3) We failed to assess the psychological components, which could affect motivation and performance. We recommend that researchers in future studies consider assessments such as internal load (e.g., HR parameters) and external load (e.g., high-intensity running variables, accelerometer variables with the Global Positioning System) for load monitoring. Additionally, assessment of factors such as skeletal age can be used to detect puberty and, ultimately, researchers could consider hormones related to puberty such as testosterone, growth hormone, and IGF-1.

## 5. Conclusions

The main novelty of this study was to predict the percentage of changes in performance ability with adjustments to the training load parameters, using multivariate regression analysis, while considering PHV and maturity offset. The present study showed the effect of changes in training load parameters and motor demand, and the most crucial correlation was that VO_2max_ in the first half was related to VO_2max_ in the second half. Aerobic variables improved during the season and had a strong relationship with PHV. Additionally, the interaction between the variables determining premature puberty and higher performance in elite soccer athletes showed a significant difference. The early and second half of the season saw notable changes, and the season-long degree of preparation (i.e., increasing VO_2max_) of young players varied significantly. This study offers new valuable insights into the practice of accumulation and motor demand in elite young players. Based on these results, coaches can consider possible differences in performance and understand the pressure of training among players in the same age group by evaluating the players’ maturity.

## Figures and Tables

**Table 1:** Matches and training sessions during the season.

Variables	Before League	1st Half	2nd Half	After League	Total
Weeks (n)	1	8	7	1	17
Training sessions (n)	4	30	23	2	59
Matches (n)	-	8	8	-	16

**Table 2:** Correlations between the maturity offset workload parameters and locomotor demand.

Variable	MO	VO_2max_1	VO_2max_2	AWL1	AWL2	CWL1	CWL2	ACWR1	ACWR2	TM1	TM2	TS1	TS2
MO	1												
VO_2max_1	−0.06	1											
VO_2max_2	−0.19	0.85 **	1										
AWL1	0.02	−0.03	−0.06	1									
AWL2	0.12	0.12	0.32	0.05	1								
CWL1	0.14	0.37	0.29	0.19	0.13	1							
CWL2	0.01	−0.01	0.13	−0.08	0.56	−0.08	1						
ACWR1	−0.05	−0.08	−0.02	−0.25	−0.13	−0.59 **	−0.06	1					
ACWR2	0.11	0.05	0.14	0.03	−0.05	0.18	0.41 *	−0.13	1				
TM1	0.17	−0.01	−0.13	0.47 *	0.01	0.36	0.05	−0.29	0.23	1			
TM2	−0.03	−0.23	−0.38	0.09	0.04	0.09	−0.08	−0.10	−0.03	0.04	1		
TS1	0.19	−0.09	−0.26	0.48	0.05	0.40 *	0.01	−0.37 *	0.19	0.96 **	0.04	1	
TS2	0.06	0.13	0.35	0.07	0.16	−0.13	0.51 **	−0.17	−0.01	−0.01	0.01	0.16	1

AWL = the accumulated acute workload in the season; CWL= the accumulated chronic workload in the season; ACWR = the accumulated acute: chronic workload ration in the season; TM = the accumulated training monotony in the season; TS = the accumulated training strain in the season; PHV = Peak height velocity 1: 1st half of the season; 2: 2nd half of the season; VO_2max_: maximum rate of oxygen consumption. * *p* < 0.05; ** *p* < 0.01.

**Table 3:** Correlations between the peak height velocity, workload parameters, and locomotor demand.

Variable	PHV	VO_2max_1	VO_2max_2	AWL1	AWL2	CWL1	CWL2	ACWR1	ACWR2	TM1	TM2	TS1	TS2
PHV	1												
VO_2max_1	−0.01	1											
VO_2max_2	0.04	0.85 **	1										
AWL1	−0.07	−0.12	0.11	1									
AWL2	−0.09	−0.01	0.10	0.04	1								
CWL1	−0.07	0.36	0.28	0.22	0.10	1							
CWL2	0.12	0.20	0.23	−0.07	0.62 **	−0.09	1						
ACWR1	−0.08	−0.06	−0.21	−0.29	−0.08	−0.53 **	−0.06	1					
ACWR2	−0.03	−0.03	−0.05	0.05	0.83 **	0.15	0.49 **	−0.18	1				
TM1	0.01	−0.11	−0.09	−0.54 **	−0.03	0.32 *	0.05	−0.29	0.05	1			
TM2	0.08	−0.18	−0.21	−0.06	0.03	0.16	−0.09	−0.10	−0.01	0.04	1		
TS1	−0.17	−0.05	−0.11	0.34 *	−0.12	0.54 **	−0.13	0.34 *	0.08	0.02	0.16	1	
TS2	−0.03	0.02	0.18	0.18	0.34 *	−0.12	0.51 **	−0.18	0.34	0.09	0.00	−0.16	1

AWL = the accumulated acute workload in the season; CWL= the accumulated chronic workload in the season; ACWR = the accumulated acute: chronic workload ration in the season; TM = the accumulated training monotony in the season; TS = the accumulated training strain in the season; PHV = Peak height velocity 1: 1st half of the season; 2: 2nd half of the season; VO_2max_: maximum rate of oxygen consumption. * *p* < 0.05; ** *p* < 0.01.

**Table 4:** Percentage of change in aerobic power with workload and maturity offset using multiple linear regression analysis.

Variables	Beta	Estimate	|t|	*p*	Estimate (95% CI)	TP
VO_2max_ (%)	β0	−8.258	0.79	0.44	−30.5. 13.9	R^2^: 0.46 Estimated R^2^: 0.35 *p*: 0.02 AIC value: 30.67
CWL (A.U.)	β1	−0.125	0.20	0.84	−0.001. 0.001	
TM (A.U.)	β2	−0.358	1.60	0.13	−0.83. 0.11	
Maturity offset (yr)	β3	0.917	1.35	0.19	−0.53. 2.36	

CWL= the accumulated chronic workload in the season; TM = the accumulated training monotony in the season; VO_2max_: maximum rate of oxygen consumption; % = the percentage of change in between assessments from early-season to after-season; AIC: Akaike information criterion. and CI = confidence interval.

**Table 5:** Percentage of change in aerobic power with workload and PHV using multiple linear regression analysis.

Variables	Beta	Estimate	|t|	*p*	Estimated (95% CI)	TP
VO_2max_ (%)	β0	6.224	1.580	0.13	−2.172. 14.6	R^2^: 0.41 Estimated R^2^: 0.28 *p*: 0.045 AIC value: 32.68
ACWR (A.U.)	β1	−0.201	0.51	0.61	−1.025. 0.623	
TM (A.U.)	β2	−0.355	2.41	0.02	−0.669. 0.041	
PHV (yr)	β3	−0.145	0.22	0.82	−1.50. 1.21	

ACWR= the accumulated acute: chronic workload ration in the season; TM = the accumulated training monotony in the season; PHV = peak height velocity; % = the percentage of change in between assessments from early-season to after-season; AIC: Akaike information criterion. and CI = confidence interval; VO_2max_: maximum rate of oxygen consumption.

## Data Availability

The data presented in this study are available on reasonable request to the corresponding author. The data are not publicly available due to privacy reasons.
